# Clinical and Molecular Characterization of Human *Burkholderia mallei* Infection, Brazil

**DOI:** 10.3201/eid3011.240549

**Published:** 2024-11

**Authors:** Kleber G. Luz, Fernanda R.O. Bezerra, Miguel A. Sicolo, Anuska A.R.S. Silva, Andréa A. Egito, Paula A.P. Suniga, Jessica C.K. Moriya, Maria G. Santos, Cynthia Mantovani, Júlia S. Silva, Nalvo F. Almeida, Ana Marcia S. Guimarães, Alberto M.R. Dávila, Rodrigo Jardim, Lenita R. Santos, Flábio R. Araújo

**Affiliations:** Universidade Federal do Rio Grande do Norte, Natal, Brazil (K.G. Luz); Universidade Potiguar, Natal (F.R.O. Bezerra); Casa de Saúde São Lucas, Natal (M.A. Sicolo); Laboratório DNA Center, Natal (A.A.R.S. Silva); Embrapa Beef Cattle, Campo Grande, Brazil (A.A. Egito, P.A.P. Suniga, J.C.K. Moriya, M.G. Santos, C. Mantovani, J.S. Silva, L.R. Santos, F.R. Araújo); Universidade Federal de Mato Grosso do Sul, Campo Grande (N.F. Almeida); University of São Paulo, São Paulo, Brazil (A.M.S. Guimarães), Oswaldo Cruz Institute, FIOCRUZ, Rio de Janeiro, Brazil (A.M.R. Dávila, R. Jardim)

**Keywords:** *Burkholderia mallei*, glanders, genome sequencing, PCR, microbiological culture, bacteria, zoonoses, Brazil

## Abstract

We report a case of *Burkholderia mallei* causing glanders in a 73-year-old patient from the Northeast Region of Brazil. The patient was hospitalized with severe pneumonia. PCR and genomic sequencing confirmed *B. mallei* in pleural drainage. Genotyping revealed a novel genotype, emphasizing the need for genetic surveillance in zoonotic infections.

*Burkholderia mallei* is a gram-negative bacterium that causes glanders disease, which primarily affects equids. *B. mallei* can infect humans and cause clinical manifestations ranging from subclinical infections to severe conditions such as septicemia or pneumonia. Treatment and prevention are difficult because of antimicrobial resistance, intracellular survival, and lack of a vaccine (*1*). In Brazil, *B. mallei* infections in equids have occurred across various regions (*2*–*6*). However, genotyping studies of *B. mallei* strains are limited. In the Northeast Region of Brazil, strains with lineages and branches L3B2, L3B3, and L3B2 have been identified. In the Southeast Region of Brazil, genotype L3B2 has been reported (*2*,*4*,*5*).

Surveillance and scientific understanding of human *B. mallei* infection in Brazil remain limited. *B. mallei* was identified in a case involving a child from the Northeast Region of Brazil (*7*). However, that publication did not specify the diagnostic methodology used to confirm the pathogen’s identity. Accurate diagnostics are crucial because melioidosis caused by *B*. *pseudomallei*, which can lead to similar clinical and pathological outcomes, is also found in Brazil (*8*).

We report a case of *B. mallei* infection in a patient from Brazil. This case provides insights into the probable transmission context, clinical symptoms, treatment approaches, and methods for detecting *B. mallei*.

## The Study

**Figure 1:**
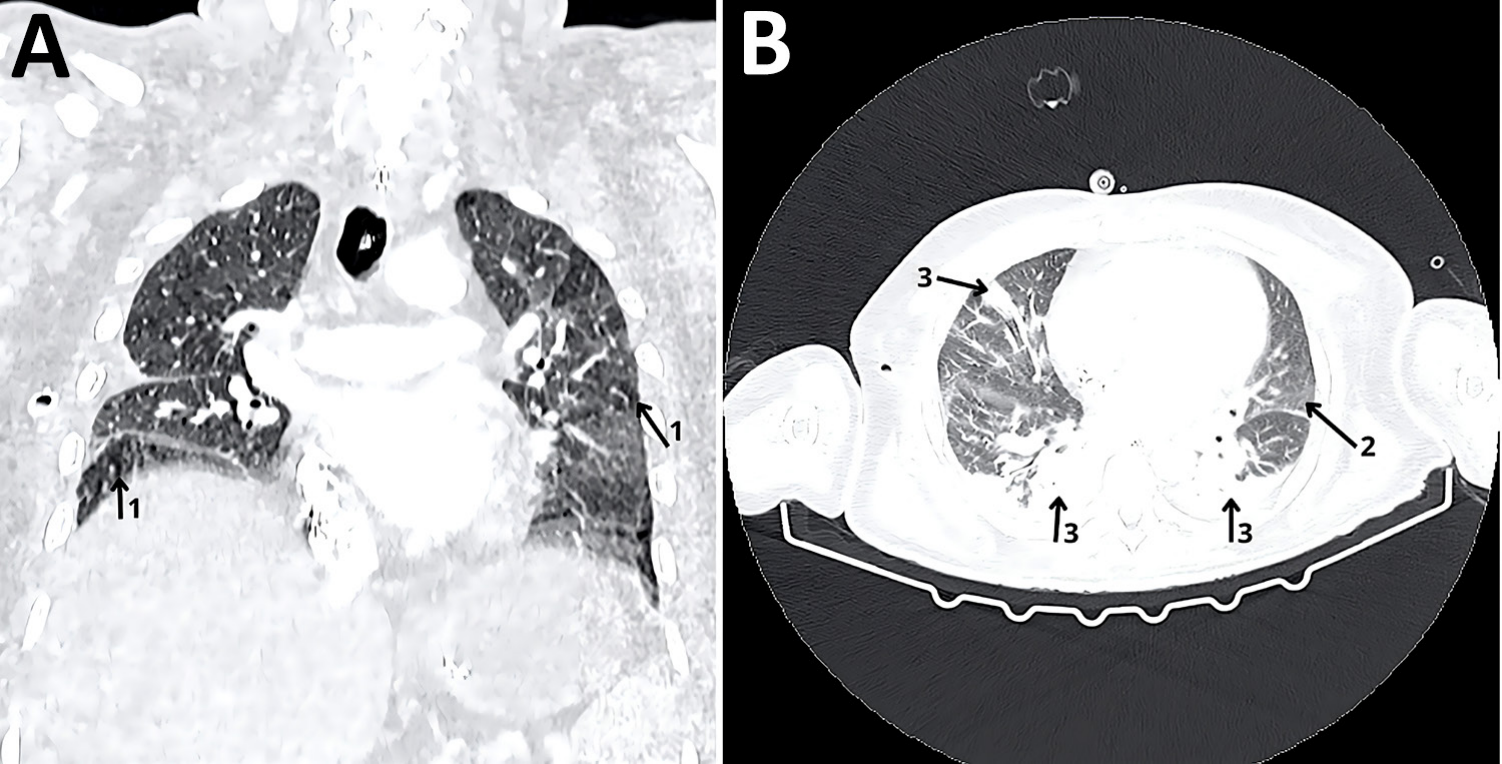
Computed tomography with contrast of the chest of a patient from Brazil infected with *Burkholderia mallei*. A) Coronal section, showing a heterogeneous pattern of lung attenuation. Arrows marked 1 indicate a suggestive disturbance in the ventilation-perfusion relationship. B) Axial section. Arrow marked 2 indicates consolidative opacities. Arrows marked 3 indicate subsegmental atelectasis in both lungs.

A 73-year-old man residing in Natal, Rio Grande do Norte, northeast Brazil, was hospitalized with complaints of fever and respiratory symptoms (Appendix). The patient’s medical history revealed his horse was in contact with a glanders-positive horse at a vaquejada training center. In the Northeast Region, equestrian events bring together horses from various sources, which increases the risk for *B. mallei* transmission and infection. Close interactions between horse owners and horses raise the likelihood of human exposure to infected animals. After 6 days of hospitalization, the patient underwent a computed tomography of his chest (Figure 1). On day 7 of hospitalization, a 25-mL sample of pleural drainage was collected and transported to the Embrapa Beef Cattle Biosafety Level 3 laboratory in Campo Grande, Brazil.

We extracted DNA from the pleural drainage by using a DNeasy Blood & Tissue Kit (QIAGEN, https://www.qiagen.com). We conducted microbiome DNA enrichment by using the NEBNext microbiome DNA enrichment kit (New England Biolabs, https://www.neb.com). We inoculated 100 μL of pleural drainage onto 5% sheep blood agar with 2% glycerol and incubated aerobically at 37°C for 24–72 hours. We cultured 100 μL of pleural drainage in 3 mL of brain heart infusion medium with 2% glycerol, with and without penicillin G and polymyxin B, and incubated at 37°C with shaking for 24 hours. From each brain–heart infusion culture, we plated 50 μL on glycerinated blood agar and incubated at 37°C with shaking for 24 hours.

We subcultured colonies matching the macroscopic identification of *B. mallei* on semi-selective agar with penicillin G (100 U/mL), polymyxin B (50 U/mL), disodium ticarcillin (32 μg/mL), ampicillin (32 μg/mL), and trimethoprim/sulfamethoxazole (TMP/SMX) (50 μg/mL + 10 μg/mL), and incubated for <72 hours. The pleural drainage culture initially grew slowly on glycerinated blood agar. Subsequent plating on semiselective agar and agar without antimicrobial drugs led to the isolation of colonies with consistent morphology. We chose a single colony and cultivated on glycerinated blood agar, yielding multiple colonies with identical morphology. We then collected the colonies for DNA extraction and PCR analysis.

We extracted DNA from bacterial isolates by using a modified protocol (*9*). Conventional PCR targeted specific genetic loci from pleural drainage and bacterial isolates (Appendix Table 1). Each PCR reaction included a negative control and a positive control (*B. mallei* DNA, strain BAC 86/19) (*5*). We included PCR extraction controls (parallel DNA extraction of *E. coli*) in assays targeting fliP-IS407A (Appendix Table 1) with DNA from pleural drainage and fliP-IS407A (Appendix Table 1) with DNA from bacterial colonies. PCR conducted by using DNA extracted directly from bacterial colonies included control DNA from a field strain of *B. pseudomallei* supplied by the Ceará Central Laboratory, Brazil. We included the control DNA in reactions targeting the multiple-locus variable number tandem repeat analysis marker Bm17. We analyzed PCR products by using gel electrophoresis and whole-genome sequencing (Appendix).

**Figure 2:**
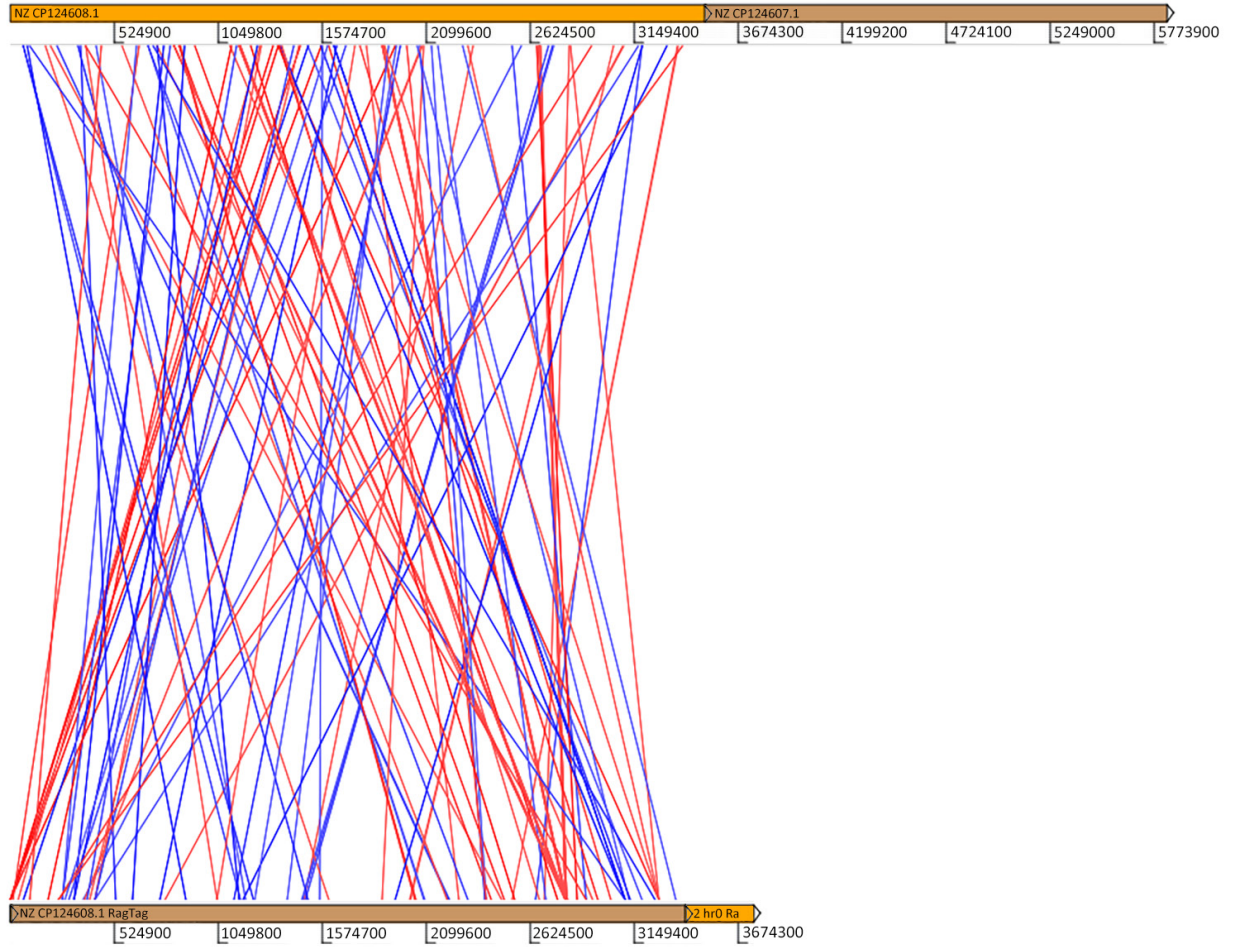
Synteny graph between the assembled genome (bottom) and the reference genome (top) of *Burkholderia mallei* recovered from a patient in Brazil. The lines represent regions of similarity; blue lines indicate sense and red lines antisense.

PCR amplifications targeting *B. mallei* loci (Appendix Table 1) from pleural drainage and bacterial isolate DNA yielded positive results. In addition, Burk475 PCR, which is designed to detect both *B. mallei* and *B. pseudomallei*, demonstrated positive amplification. The PCR targeting the open reading frame 11 marker for *B. pseudomallei* was negative (Figures 1, 2; Appendix). The amplicon sequencing results (Appendix Table 2) confirmed exact matches with *B. mallei* from PCR targeting the species.

The bacterial genome sequencing revealed 5,506,149 reads (National Center for Biotechnology Information Sequence Read Archive accession no. PRJNA1130892). De novo assembly produced 881 contigs, with an N50 of 2,605,114 bp. Synteny analysis indicated 112 regions shared similarity with *B. mallei* (Figure 2). BLASTX analysis (https://blast.ncbi.nlm.nih.gov) showed 110 contigs (12.48%) matched both *B. mallei* and *B. pseudomallei* with 100% identity, whereas 59 contigs (6.69%) specifically matched *B. mallei*, differing from *B. pseudomallei* because of single-nucleotide polymorphisms (SNPs), insertions, or deletions (Appendix Table 3).

Analysis identified an SNP at position 1,163,826 in the reference genome of *B. mallei*. In the human-origin isolate studied (*B. mallei* Natal strain), this SNP manifested as a T allele, a characteristic feature observed in isolates belonging to the L2B2sB1Gp1 lineage.

## Conclusions

We successfully detected *B. mallei* in this patient by using various PCR targets, directly from both pleural drainage and bacterial cultures. The methodology included culturing a limited number of bacterial colonies from the sample, followed by subculturing to various media and antimicrobial drug conditions. Necessary to the process was the careful selection of colonies on the basis of consistent morphological characteristics, which was essential for the subsequent PCR detection.

The presence of a limited number of contigs showing identity with *Burkholderia* species in the genome sequencing of the PCR-positive colony suggests the potential coculturing of competing microbiota. However, 59 contigs showed exact matches with *B. mallei*, distinguishing them from *B. pseudomallei* because of variations such as SNPs, insertions, or deletions. Those matches aligned consistently with the reference *B. mallei* genome and were validated across multiple other *B. mallei* genomes, confirming the presence of this species in the sample through genome sequencing.

A SNP characteristic of isolates from the L2B2sB1Gp1 lineage was identified in the *B. mallei* Natal strain. This lineage includes strains from the United States and Burma (ATCC 23344) from humans and strains from Myanmar and China from equids (*10*). Of note, in Brazil only isolates from lineage 3 have been reported, all from equids (*2*,*4*,*5*).

Treating glanders disease is challenging because of *B. mallei*’s resistance to many common antimicrobial drugs. A recent case from China highlighted initial therapy failure with levofloxacin and cefotaxime/sulbactam, but success was achieved by adding meropenem, doxycycline, and TMP/SMX (*11*). In Iran, effective treatment involved imipenem and doxycycline (*12*), aligning with established guidelines for managing zoonotic glanders (*13*). In this case, initial treatment with ceftriaxone followed by meropenem and azithromycin did not improve symptoms. However, combining meropenem, linezolid, TMP/SMX, and levofloxacin resulted in improvement in the patient’s condition. 

Human glanders disease is likely underreported and underrecognized, underscoring the importance of increased awareness among medical professionals. The use of effective antimicrobial drugs is necessary for patient treatment. Management of human *B. mallei* infections requires enhanced coordination between veterinary and human health specialists.

Appendix Additional information about clinical and molecular characterization of human *Burkholderia mallei* infection, Brazil.
