# Supplementary material for: Clinical and Molecular Characterization of Human Burkholderia mallei Infection, Brazil
Source: Emerg Infect Dis. 2024 Nov;30(11):2400–3. doi: 10.3201/eid3011.240549 (PMC11521173; doi:10.3201/eid3011.240549)
Supplement: Appendix — Additional information about clinical and molecular characterization of human Burkholderia mallei infection, Brazil. [file 24-0549-Techapp-s1.pdf]

Article DOI: https://doi.org/10.3201/eid3011.240549

EID cannot ensure accessibility for supplementary materials supplied by authors. Readers who have difficulty accessing supplementary content should contact the authors for assistance.

Clinical and Molecular Characterization of Human *Burkholderia mallei* Infection, Brazil

Appendix

Whole Genome Sequencing

Libraries were prepared with the NEBNext® Ultra II DNA Library Prep Kit (New England Biolabs, MA, USA). Suitable libraries were pooled and sequenced with the NovaSeq6000 (Illumina, San Diego, CA, USA) using the NovaSeq 6000 S4 Reagent Kit v1.5 (paired end, 300 cycles).

The sequenced data underwent quality assessment using FASTQC (v0.11.9) and trimming with TRIMMOMATIC (v0.39), adhering to specified criteria (PHRED quality >28, minimum length >28, and adaptor removal). De novo assembly was performed using SPADES (v3.15.5) (*1*), and scaffolding was achieved with RAGTAG (v2.1.0) (*2*) using the *B. mallei* reference genome (ATCC 23344, GCF_033956065.1_ASM3395606v1). Synteny analysis was conducted using the ACT software (v18.0.0) (*3*) against the *B. mallei* reference genome, followed by extraction of syntenic regions in fasta format. These regions were then subjected to blastX (v2.12.0) against the REFSEQ database (release 222) (*4*) to annotate the functional aspects of identified *B. mallei* genes in the assembly.

The identified hits underwent blastN analysis against the nr database, and the initial 100 matches were scrutinized. Contigs exhibiting identity solely with either *B. mallei* or *B. pseudomallei* were subsequently subjected to blastN against the reference genomes of *B. mallei* and *B. pseudomallei* (strain K96243, GCA_000959285.1_ ASM75612v1).

Clinical presentation of the patient with glanders

On July 23, 2023, the patient presented with symptoms including cough, fever, malaise, chest pain, and arterial hypertension. Clinical examination indicated pneumonia, and treatment commenced with intravenous ceftriaxone 2g/day upon admission. Lung auscultation revealed decreased vesicular sounds in the right base, accompanied by crepitations in the same region.

Chest radiography identified opacity in the right base, while computed tomography (CT) revealed opacity in the right middle and lower lobes accompanied by moderate pleural effusion. Laboratory results indicated elevated C-reactive protein (CRP) levels of 328 mg/dL and leukocytosis with neutrophilia (14,000 cells/mm^3). The working diagnosis was pneumonia with secondary elevated troponin, consistent with pleuritic chest pain (type D). Consequently, the patient was admitted to the hospital and initiated on intravenous meropenem 1g every six hours.

On the second day, the patient presented with tachypnea and persistent fever. Lung auscultation revealed crepitations in the lung bases and diffuse rhonchi. Azithromycin 500 mg intravenously, once daily, was initiated. On the third day, during the medical history-taking session, the patient disclosed that they own a horse used in *vaquejada*, a classic Brazilian equestrian competition. He noted that his horse shared facilities at a *vaquejada* training center with a mare, which subsequently tested positive for glanders in serologic testing. Pulmonary consolidation in the right base and peripheral cyanosis were observed. The patient was transferred to the Intensive Care Unit (ICU), subjected to blood and urine cultures (all negative), and prescribed meropenem 1g intravenously every six hours and linezolid 600 mg intravenously twice daily. The patient developed rhonchi and crepitations in the right hemithorax, with ultrasound revealing a 350 mL effusion and a saturation of 91%.

On the fifth day, trimethoprim/sulfamethoxazole (TMP-SMX) 800/80 mg was initiated intravenously every six hours, pending culture results, and the patient recorded a fever of 37.9°C. On the sixth day, leukocytosis persisted, with a count of 14,680 cells/mm^3. Subsequently, on the sixth day, the patient developed respiratory failure, prompting a chest CT which revealed consolidations in the lower lobes and fine opacities in the middle lobe, associated with cylindrical bronchiectasis in the middle lobe and lower lobes. Moderate right-sided pleural effusion, and small left-sided pleural effusion. These findings were consistent with consolidations in the middle lobe and lower right lobe, associated with cylindrical bronchiectasis

in the middle lobe and lower lobes, with moderate bilateral pleural effusion. Pleural drainage was performed on the seventh day, yielding citrine fluid with increased protein but without cells. Additionally, reduced urine output and worsening renal function were noted, with a creatinine level of 1.7 mg/dL. Due to respiratory failure, the patient was intubated. On the eighth day, nephrology evaluation confirmed renal failure with a creatinine level of 2.3 mg/dL. Levofloxacin 500 mg/day intravenously was prescribed, and adrenaline was administered due to fever (38°C) and leukocytosis (10,390 cells/mm^3). On the tenth day, leukocyte count was 14,230 cells/mm^3, and creatinine was 1.8 mg/dL. No fever was recorded, and pleural fluid culture was negative for acid-fast bacilli (tuberculosis). The patient continued on hemodialysis. Leukocyte count decreased to 12,630 cells/mm^3 on the eleventh day, followed by a further decrease to 6,110 cells/mm^3, with a CRP level of 48.2 mg/dL on the thirteenth day. On the fourteenth day, the patient remained subfebrile and hemodynamically stable, with a creatinine level of 0.8 mg/dL. Extubation was performed on this day, with subsequent days showing no fever, leukocytes of 8,500 cells/mm^3, creatinine of 0.9 mg/dL, and CRP of 59 mg/dL. On the sixteenth day, the patient continued treatment with meropenem, TMP-SMX, and levofloxacin. On the seventeenth day, linezolid and meropenem were discontinued. With the resolution of septic shock, the patient was discharged from the ICU on the twentieth day and later discharged from the hospital.

References

1. Prjibelski A, Antipov D, Meleshko D, Lapidus A, Korobeynikov A. Using SPAdes de novo assembler. Curr Protoc Bioinformatics. 2020;70:e102. PubMed https://doi.org/10.1002/cpbi.102

2. Alonge M, Lebeigle L, Kirsche M, Jenike K, Ou S, Aganezov S, et al. Automated assembly scaffolding using RagTag elevates a new tomato system for high-throughput genome editing. Genome Biol. 2022;23:258. PubMed https://doi.org/10.1186/s13059-022-02823-7

3. Carver TJ, Rutherford KM, Berriman M, Rajandream MA, Barrell BG, Parkhill J. ACT: the artemis comparison tool. Bioinformatics. 2005.21(16):3422–3

4. O'Leary NA, Wright MW, Brister JR, Ciufo S, Haddad D, McVeigh R, et al. Reference sequence (RefSeq) database at NCBI: current status, taxonomic expansion, and functional annotation. Nucleic Acids Res. 2016;44(D1):D733–45. PubMed https://doi.org/10.1093/nar/gkv1189

5. Scholz HC, Joseph M, Tomaso H, Al Dahouk S, Witte A, Kinne J, et al. Detection of the reemerging agent *Burkholderia mallei* in a recent outbreak of glanders in the United Arab Emirates by a newly developed fliP-based polymerase chain reaction assay. Diagn Microbiol Infect Dis. 2006;54:241–7. PubMed https://doi.org/10.1016/j.diagmicrobio.2005.09.018

6. Abreu DC, Gomes AS, Tessler DK, Chiebao DP, Fava CD, Romaldini AHCN, et al. Systematic monitoring of glanders-infected horses by complement fixation test, bacterial isolation, and PCR. Vet Anim Sci. 2020;10:100147. PubMed https://doi.org/10.1016/j.vas.2020.100147

7. Fonseca Júnior AA, Pinto CA, Alencar CAS, Bueno BL, Dos Reis JKP, de Carvalho Filho MB. Validation of three qPCR for the detection of *Burkholderia mallei* in equine tissue samples. Arch Microbiol. 2021;203:3965–71. PubMed https://doi.org/10.1007/s00203-021-02367-7

8. Aung NM, Su KK, Chantratita N, Tribuddharat C. Workflow for identification of *Burkholderia pseudomallei* clinical isolates in Myanmar. Jpn J Infect Dis. 2023;76:106–12. PubMed https://doi.org/10.7883/yoken.JJID.2022.508

9. Wong Su Yen M, Lisanti O, Thibault F, Su San T, Gek Kee L, Hilaire V, et al. Validation of ten new polymorphic tandem repeat loci and application to the MLVA typing of *Burkholderia pseudomallei* isolates collected in Singapore from 1988 to 2004. J Microbiol Methods. 2009;77:297–301. PubMed https://doi.org/10.1016/j.mimet.2009.03.005

Appendix Table 1. Primers employed in the study.

Target locus	Sequence (5` to 3`)	Amplicon size (pb)	Target species	Reference
fliP-IS407A (a)	F:TCAGGTTTGTATGTCGCTCGG<br>R: CTAGGTGAAGCTCTGCGCGAG	989	*B. mallei*	(*5*)
fliP-IS407A (b)	F: TCAGGTTTGTATGTCGCTCGG<br>R: GCCCGACGAGCACCTGATT	528	*B. mallei*	(*6*)
Type IV secretion protein Rhs (Burk475)	F: AATTGGTCGCCGTGATAGAC<br>R: TCGGCTCGATGGAAAGTAAC	475	*B. mallei*<br>*B. pseudomallei*	(*7*)
orf11	F: ATCGCCAAATGCCGGGTTTC<br>R: GTGCATCCATTCATCAAAG	706	*B. pseudomallei*	(*8*)
MLVA marker (Bm17)	F: TATACGCGAGGTTATAACGGATG<br>R: CTTTCTGCTTTTCTAACGTTTCC	281<br>321	*B. mallei*<br>*B. pseudomallei*	(*9*)

Appendix Table 2. Analysis of BlastN Amplicon Sequencing Results from Pleural Drainage DNA and Bacterial Isolates.

Sample identification	PCR target	Best hit	E-value	Identity
Pleural drainage	fliP-IS407A (*6*)	*Burkholderia mallei fliP pseudogene, partial sequence; and IS407A transposase (tnpB) gene, partial cds*	0.0	100%
Pleural drainage	MLVA marker Bm17 (*9*)	*Burkholderia mallei strain Turkey10 chromosome 2, complete sequence*	$2{,}00^{-75}$	92.75%
Pleural drainage bacterial isolate	fliP-IS407A (*6*)	*Burkholderia mallei strain Turkey 10 clone 1–6.6 fliP mobile element, partial sequence*	0.0	100%
Pleural drainage bacterial isolate	MLVA marker Bm17 (*9*)	*Burkholderia mallei strain Turkey10 chromosome 2, complete sequence*	$7{,}00^{-136}$	100%
Pleural drainage bacterial isolate	type IV secretion protein Rhs - Burk475 (*7*)	*Burkholderia mallei strain Turkey10 chromosome 2, complete sequence*	0.0	100%

Appendix Table 3. Results of homology searches in BLASTn (NCBI) for contigs assembled from genomic DNA sequencing from human patient pleural aspirate cultures. The sequences were compared with references of *Burkholderia mallei* ATCC 23344 and *Burkholderia pseudomallei* K96243.

	Burkholderia mallei ATCC 23344				***Burkholderia pseudomallei K96243***			
**Contig number**	**Position in the reference genome**	**Query Cover**	**E-value**	**Identity**	**Position in the reference genome**	**Query Cover**	**E-value**	**Identity**
assembly_1_279|ref_1326310_1326588|100|length:278	1326335 to 1326612	100%	2,00E-146	100.00%	3895064 to 3895341	100%	5,00E-141	98.92%
assembly_380_610|ref_1352787_1353017|100|length:230	1352812 to 1353041	100%	8,00E-120	100.00%	1804504 to 1804733	100%	9,00E-118	99.57%
					637361 to 637553	83%	8,00E-54	86.01%
assembly_711_978|ref_1467879_1468146|100|length:267	1467906 to 1468172	100%	2,00E-140	100.00%	1684787 to 1684890	38%	1,00E-47	99.04%
assembly_1079_1332|ref_1568224_1568477|99|length:253	1568251 to 1568503	100%	1,00E-132	100.00%	2546598 to 2546850	100%	2,00E-130	99.60%
assembly_1433_1663|ref_1942844_1943074|100|length:230	1942871 to 1943100	100%	8,00E-120	100.00%	1375128 to 1375357	100%	2,00E-119	100.00%
assembly_16329_17967|ref_653645_655280|81|length:1635	653677 to 655311	100%	0.0	100.00%	2849845 to 2851479	100%	0.0	99.94%
assembly_41071_41332|ref_1260373_1260634|74|length:261	1260398 to 1260658	100%	5,00E-137	100.00%	2129702 to 2129962	100%	1,00E-136	100.00%
assembly_69628_69596|ref_1508879_1508911|96|length:32	1508906 to 1508937	100%	6,00E-11	100.00%	1644002 to 1644033	100%	1,00E-10	100.00%
assembly_100621_100837|ref_461290_461506|78|length:216	461322 to 461537	100%	4,00E-112	100.00%	3038553 to 3038768	100%	1,00E-111	100.00%
assembly_191609_191641|ref_1119504_1119535|96|length:31	1119537 to 1119567	100%	2,00E-10	100.00%	2295525 to 2295555	100%	5,00E-10	100.00%
assembly_245680_245792|ref_3397975_3398087|83|length:112	3398038 to 3398149	100%	1,00E-54	100.00%	246870 to 246981	100%	2,00E-52	99.11%
assembly_247304_247535|ref_2933800_2934029|79|length:229	2933792 to 2934020	100%	3,00E-119	100.00%	3906767 to 3906995	100%	3,00E-117	99.56%
assembly_267370_266807|ref_810297_810860|78|length:563	810329 to 810891	100%	0.0	100.00%	1583761 to 1584323	100%	0.0	99.64%
assembly_267478_266400|ref_639714_640795|74|length:1081	639746 to 640826	100%	0.0	100.00%	2864325 to 2865405	100%	0.0	99.72%
assembly_278651_278461|ref_1601332_1601522|74|length:190	1601359 to 1601548	100%	1,00E-97	100.00%	2578179 to 2578368	100%	1,00E-95	99.47%
assembly_301601_301527|ref_1696518_1696592|81|length:74	1696531 to 1696604	100%	1,00E-33	100.00%	2671914 to 2671987	100%	3,00E-33	100.00%
assembly_322413_322185|ref_775976_776204|100|length:228	776008 to 776235	100%	1,00E-118	100.00%	1200207 to 1200434	100%	2,00E-118	100.00%
assembly_322413_322185|ref_1251040_1251268|100|length:228	1251065 to 1251292	100%	1,00E-118	100.00%	2120411 to 2120638	100%	2,00E-118	100.00%
assembly_370119_369739|ref_2579660_2580040|76|length:380	2579679 to 2580058	100%	0.0	100.00%	3532250 to 3532629	100%	0.0	99.47%

	Burkholderia mallei ATCC 23344				*Burkholderia pseudomallei K96243*			
**Contig number**	**Position in the reference genome**	**Query Cover**	**E-value**	**Identity**	**Position in the reference genome**	**Query Cover**	**E-value**	**Identity**
assembly_377422_379099|ref_2079770_2081444|70|length:1674	2079789 to 2081462	100%	0.0	100.00%	3204685 to 3206358	100%	0.0	99.64%
assembly_393582_393507|ref_1144423_1144498|80|length:75	1144456 to 1144530	100%	3,00E-34	100.00%	2320527 to 2320601	100%	7,00E-34	100.00%
assembly_403334_403579|ref_1457837_1458080|74|length:243	1457864 to 1458106	100%	5,00E-127	100.00%	1699227 to 1699469	100%	1,00E-126	100.00%
assembly_431021_431337|ref_1270619_1270935|72|length:316	1270644 to 1270959	100%	2,00E-167	100.00%	2190238 to 2190553	100%	4,00E-167	100.00%
assembly_431371_431242|ref_1431184_1431313|79|length:129	1431209 to 1431337	100%	6,00E-64	100.00%	1726017 to 1726145	100%	1,00E-58	97.67%
assembly_431662_431081|ref_3173146_3173730|73|length:584	3173208 to 3173791	100%	0.0	100.00%	1514241 to 1514824	100%	0.0	99.83%
assembly_532668_532617|ref_356012_356062|92|length:50	356044 to 356093	100%	1,00E-20	100.00%	967570 to 967619	100%	3,00E-20	100.00%
assembly_621028_620999|ref_880853_880882|100|length:29	880885 to 880913	100%	2,00E-09	100.00%	2467367 to 2467395	100%	5,00E-09	100.00%
assembly_754853_754824|ref_1648332_1648361|100|length:29	1648359 to 1648387	100%	2,00E-09	100.00%	2625315 to 2625343	100%	5,00E-09	100.00%
assembly_782683_783959|ref_3096390_3097696|74|length:1306	3096382 to 3097687	100%	0.0	100.00%	3988016 to 3989321	100%	0.0	100.00%
assembly_885894_885863|ref_90076_90107|96|length:31	90101 to 90131	100%	2,00E-10	100.00%	418580 to 418610	100%	5,00E-10	100.00%
assembly_1047137_1047451|ref_1740831_1741145|73|length:314	1740844 to 1741157	100%	2,00E-166	100.00%	2716305 to 2716618	100%	5,00E-166	100.00%
assembly_1047141_1047427|ref_384946_385235|77|length:289	384978 to 385266	100%	2,00E-152	100.00%	996632 to 996920	100%	9,00E-149	99.31%
assembly_1131055_1130996|ref_1974130_1974189|86|length:59	1974157 to 1974215	100%	2,00E-25	100.00%	1345232 to 1345290	100%	4,00E-25	100.00%
assembly_1155366_1155338|ref_743717_743745|100|length:28	743749 to 743776	100%	7,00E-09	100.00%	1169545 to 1169572	100%	2,00E-08	100.00%
assembly_1166622_1166547|ref_71214_71287|83|length:73	71239 to 71311	100%	4,00E-33	100.00%	437468 to 437540	100%	9,00E-33	100.00%
assembly_1177718_1177684|ref_3316585_3316619|94|length:34	3316647 to 3316680	100%	5,00E-12	100.00%	1227278 to 1227311	100%	1,00E-11	100.00%
assembly_1190126_1189518|ref_437581_438190|74|length:609	437613 to 438221	100%	0.0	100.00%	3016356 to 3016964	100%	0.0	100.00%
assembly_1232035_1231987|ref_71218_71268|90|length:50	71243 to 71292	100%	1,00E-20	100.00%	437487 to 437536	100%	3,00E-20	100.00%
assembly_1238289_1237886|ref_1952664_1953070|72|length:406	1952691 to 1953096	100%	0.0	100.00%	1366452 to 1366857	100%	0.0	99.01%

	Burkholderia mallei ATCC 23344				*Burkholderia pseudomallei K96243*			
**Contig number**	**Position in the reference genome**	**Query Cover**	**E-value**	**Identity**	**Position in the reference genome**	**Query Cover**	**E-value**	**Identity**
assembly_1402588_1402366|ref_1912156_1912378|74|length:222	1912183 to 1912404	100%	2,00E-115	100.00%	1404584 to 1404805	100%	5,00E-115	100.00%
assembly_1476499_1475673|ref_2744613_2745435|75|length:822	2744633 to 2745454	100%	0.0	100.00%	3822396 to 3823217	100%	0.0	99.76%
assembly_1479949_1479168|ref_2741128_2741909|80|length:781	2741148 to 2741928	100%	0.0	100.00%	3818911 to 3819691	100%	0.0	99.74%
assembly_1483444_1485444|ref_2335875_2337887|72|length:2012	2335886 to 2337897	100%	0.0	100.00%	1039820 to 1041831	100%	0.0	99.95%
assembly_1495200_1495100|ref_2722150_2722250|79|length:100	2722170 to 2722269	100%	6,00E-48	100.00%	3801245 to 3801344	100%	1,00E-47	100.00%
assembly_1506753_1506716|ref_346265_346302|92|length:37	346297 to 346333	100%	1,00E-13	100.00%	957834 to 957870	100%	3,00E-13	100.00%
assembly_1561824_1561527|ref_1579036_1579333|74|length:297	1579063 to 1579359	100%	6,00E-157	100.00%	2556098 to 2556394	100%	3,00E-153	99.33%
assembly_1608767_1609003|ref_178275_178511|72|length:236	178307 to 178542	100%	4,00E-123	100.00%	198035 to 198270	100%	9,00E-123	100.00%
assembly_1879973_1880124|ref_2088393_2088544|75|length:151	2088412 to 2088562	100%	4,00E-76	100.00%	3213308 to 3213458	100%	5,00E-74	99.34%
assembly_1944149_1944117|ref_3191658_3191690|96|length:32	3191720 to 3191751	100%	6,00E-11	100.00%	598061 to 598092	100%	1,00E-10	100.00%
assembly_2020498_2021115|ref_3045766_3046358|74|length:592	3045758 to 3046349	100%	0.0	100.00%	4037703 to 4038294	100%	0.0	99.49%
assembly_2041050_2042177|ref_861063_862188|74|length:1125	861095 to 862219	100%	0.0	100.00%	2486094 to 2487218	100%	0.0	99.91%
assembly_2050390_2050435|ref_853998_854043|89|length:45	854030 to 854074	100%	7,00E-18	100.00%	2494243 to 2494287	100%	2,00E-17	100.00%
assembly_2093382_2092510|ref_700761_701636|71|length:875	700793 to 701667	100%	0.0	100.00%	1126576 to 1127450	100%	0.0	99.54%
assembly_2207177_2207538|ref_566739_567101|76|length:362	566771 to 567132	100%	0.0	100.00%	2937504 to 2937865	100%	0.0	99.72%
assembly_2217882_2217445|ref_241641_242079|79|length:438	241673 to 242110	100%	0.0	100.00%	766329 to 766766	100%	0.0	99.32%
assembly_2268117_2269100|ref_1798090_1799061|73|length:971	1798103 to 1799073	100%	0.0	100.00%	2772055 to 2773025	100%	0.0	99.69%
assembly_2316023_2315919|ref_696531_696635|82|length:104	580775 to 580878	100%	3,00E-50	100.00%	1123942 to 1124045	100%	9,00E-50	100.00%
assembly_2395264_2395203|ref_2066815_2066876|83|length:61	2066834 to 2066894	100%	1,00E-26	100.00%	1076877 to 1076937	100%	3,00E-26	100.00%

Contig number	Burkholderia mallei ATCC 23344				Burkholderia pseudomallei K96243			
	Position in the reference genome	Query Cover	E-value	Identity	Position in the reference genome	Query Cover	E-value	Identity
assembly_2403996_2404189|ref_2002025_2002218|82|length:193	2002044 to 2002236	100%	2,00E-99	100.00%	1301928 to 1302120	100%	6,00E-99	100.00%
assembly_2404133_2403666|ref_2127418_2127903|75|length:485	2127429 to 2127913	100%	0.0	100.00%	3253236 to 3253720	100%	0.0	98.35%
assembly_2431309_2431349|ref_854152_854192|92|length:40	854184 to 854223	100%	3,00E-15	100.00%	2494094 to 2494133	100%	8,00E-15	100.00%
assembly_2484848_2485508|ref_204804_205470|72|length:666	204836 to 205501	100%	0.0	100.00%	729509 to 730174	100%	0.0	99.85%
assembly_2493908_2493953|ref_936607_936652|86|length:45	936639 to 936683	100%	7,00E-18	100.00%	2397615 to 2397659	100%	2,00E-17	100.00%
assembly_2536083_2536659|ref_3371999_3372569|74|length:570	3372062 to 3372631	100%	0.0	100.00%	220803 to 221372	100%	0.0	100.00%
assembly_2655899_2655192|ref_3191768_3192475|78|length:707	3191830 to 3192536	100%	0.0	100.00%	597276 to 597982	100%	0.0	100.00%
assembly_2670581_2670875|ref_1376584_1376877|75|length:293	1376609 to 1376901	100%	1,00E-154	100.00%	1780454 to 1780746	100%	2,00E-154	100.00%
assembly_2699274_2700731|ref_2825900_2827357|74|length:1457	2825920 to 2827376	100%	0.0	100.00%	3759993 to 3761449	100%	0.0	99.66%
assembly_2706575_2705712|ref_2161931_2162794|77|length:863	2161942 to 2162804	100%	0.0	99.30%	3288061 to 3288692	100%	0.0	98.12%
assembly_2802131_2803330|ref_2672911_2674100|77|length:1189	2672930 to 2674118	100%	0.0	100.00%	1435490 to 1436678	100%	0.0	99.92%
	558670 to 559858	100%	0.0	100.00%	489072 to 490260	100%	0.0	99.92%
assembly_2802131_2803330|ref_827451_828640|77|length:1189	827483 to 828671	100%	0.0	100.00%	1600914 to 1602100	100%	0.0	99.24%
assembly_2802131_2803330|ref_1158972_1160161|77|length:1189	1159005 to 1160193	100%	0.0	100.00%	2335100 to 2335803	59%	0.0	99.72%
assembly_2803909_2804688|ref_1881905_1882676|76|length:771	1881932 to 1882702	100%	0.0	100.00%	1434271 to 1435041	100%	0.0	99.48%
	557451 to 558221	100%	0.0	100.00%	487853 to 488623	100%	0.0	99.48%
assembly_2803909_2804688|ref_2674548_2675319|76|length:771	1881932 to 1882702	100%	0.0	100.00%	1434271 to 1435041	100%	0.0	99.48%
	557451 to 558221	100%	0.0	100.00%	487853 to 488623	100%	0.0	99.48%
assembly_2803909_2804688|ref_829088_829859|76|length:771	829120 to 829890	100%	0.0	100.00%	1602549 to 1603319	100%	0.0	99.09%
assembly_2803909_2804688|ref_1160609_1161380|76|length:771	1160642 to 1161412	100%	0.0	100.00%	3720996 to 3721762	99%	0.0	99.74%
	1643054 to 1643823	100%	0.0	100.00%				

	Burkholderia mallei ATCC 23344				*Burkholderia pseudomallei K96243*			
**Contig number**	**Position in the reference genome**	**Query Cover**	**E-value**	**Identity**	**Position in the reference genome**	**Query Cover**	**E-value**	**Identity**
assembly_2805477_2806992|ref_1883646_1885171|77|length:1525	1883673 to 1885197	100%	0.0	100.00%	1431776 to 1433300	100%	0.0	99.93%
	554956 to 556480	100%	0.0	100.00%	485358 to 486882	100%	0.0	99.87%
assembly_2805477_2806992|ref_2676289_2677814|77|length:1525	1883673 to 1885197	100%	0.0	100.00%	1431776 to 1433300	100%	0.0	99.93%
	554956 to 556480	100%	0.0	100.00%	485358 to 486882	100%	0.0	99.87%
assembly_2805477_2806992|ref_830829_832354|77|length:1525	830861 to 832385	100%	0.0	100.00%	1604289 to 1605813	100%	0.0	99.87%
assembly_2805477_2806992|ref_1162350_1163875|77|length:1525	1162383 to 1163907	100%	0.0	100.00%	2031620 to 2033144	100%	0.0	99.74%
assembly_2808733_2809932|ref_2672911_2674100|77|length:1189	2672930 to 2674118	100%	0.0	100.00%	1435490 to 1436678	100%	0.0	99.92%
	558670 to 559858	100%	0.0	100.00%	489072 to 490260	100%	0.0	99.92%
assembly_2808733_2809932|ref_827451_828640|77|length:1189	827483 to 828671	100%	0.0	100.00%	1600914 to 1602100	100%	0.0	99.24%
assembly_2808733_2809932|ref_1158972_1160161|77|length:1189	1159005 to 1160193	100%	0.0	100.00%	2335100 to 2335803	59%	0.0	99.72%
assembly_2810511_2811290|ref_1881905_1882676|76|length:771	1881932 to 1882702	100%	0.0	100.00%	1434271 to 1435041	100%	0.0	99.48%
	557451 to 558221	100%	0.0	100.00%	487853 to 488623	100%	0.0	99.48%
assembly_2810511_2811290|ref_2674548_2675319|76|length:771	1881932 to 1882702	100%	0.0	100.00%	1434271 to 1435041	100%	0.0	99.48%
	557451 to 558221	100%	0.0	100.00%	487853 to 488623	100%	0.0	99.48%
assembly_2810511_2811290|ref_829088_829859|76|length:771	829120 to 829890	100%	0.0	100.00%	1602549 to 1603319	100%	0.0	99.09%
assembly_2810511_2811290|ref_1160609_1161380|76|length:771	1160642 to 1161412	100%	0.0	100.00%	3720996 to 3721762	99%	0.0	99.74%
	1643054 to 1643823	100%	0.0	100.00%				
assembly_2812079_2813594|ref_1883646_1885171|77|length:1525	1883673 to 1885197	100%	0.0	100.00%	1431776 to 1433300	100%	0.0	99.93%
	554956 to 556480	100%	0.0	100.00%	485358 to 486882	100%	0.0	99.87%
assembly_2812079_2813594|ref_2676289_2677814|77|length:1525	1883673 to 1885197	100%	0.0	100.00%	1431776 to 1433300	100%	0.0	99.93%
	554956 to 556480	100%	0.0	100.00%	485358 to 486882	100%	0.0	99.87%
assembly_2812079_2813594|ref_1162350_1163875|77|length:1525	1162383 to 1163907	100%	0.0	100.00%	2031620 to 2033144	100%	0.0	99.74%
assembly_2812079_2813594|ref_830829_832354|77|length:1525	830861 to 832385	100%	0.0	100.00%	1604289 to 1605813	100%	0.0	99.87%

	Burkholderia mallei ATCC 23344				*Burkholderia pseudomallei K96243*			
**Contig number**	**Position in the reference genome**	**Query Cover**	**E-value**	**Identity**	**Position in the reference genome**	**Query Cover**	**E-value**	**Identity**
assembly_2816907_2817231|ref_744286_744610|73|length:324	744318 to 744641	100%	6,00E-172	100.00%	1170114 to 1170437	100%	2,00E-171	100.00%
assembly_2864259_2864292|ref_2658891_2658924|97|length:33	2658910 to 2658942	100%	2,00E-11	100.00%	3608456 to 3608488	100%	4,00E-11	100.00%
assembly_2907449_2907647|ref_385108_385307|81|length:199	385140 to 385338	100%	1,00E-102	100.00%	996794 to 996992	100%	1,00E-100	99.50%
assembly_2907678_2907460|ref_2535072_2535290|84|length:218	2535091 to 2535308	100%	3,00E-113	100.00%	3488957 to 3489174	100%	9,00E-113	100.00%
assembly_2942248_2942957|ref_1110183_1110877|74|length:694	1110216 to 1110909	100%	0.0	100.00%	2286165 to 2286858	100%	0.0	99.86%
assembly_2970750_2970803|ref_1608656_1608708|87|length:52	1608683 to 1608734	100%	1,00E-21	100.00%	2585503 to 2585554	100%	3,00E-21	100.00%
assembly_3034758_3034862|ref_1766823_1766927|82|length:104	1766836 to 1766939	100%	3,00E-50	100.00%	2742294 to 2742397	100%	9,00E-50	100.00%
assembly_3105298_3105251|ref_1636059_1636106|91|length:47	1636086 to 1636132	100%	6,00E-19	100.00%	2613006 to 2613052	100%	1,00E-18	100.00%
assembly_3105304_3105276|ref_1365287_1365315|100|length:28	1365312 to 1365339	100%	7,00E-09	100.00%	1792216 to 1792243	100%	2,00E-08	100.00%
assembly_3105305_3105268|ref_1636909_1636947|94|length:38	1636936 to 1636973	100%	4,00E-14	100.00%	2613856 to 2613893	100%	1,00E-13	100.00%
assembly_3105337_3105271|ref_103860_103930|82|length:70	103885 to 103954	100%	2,00E-31	100.00%	405892 to 405961	100%	4,00E-31	100.00%
assembly_3127656_3127433|ref_2054946_2055169|76|length:223	2054965 to 2055187	100%	6,00E-116	100.00%	1088584 to 1088806	100%	7,00E-114	99.55%
assembly_3133244_3133158|ref_1573422_1573508|88|length:86	1573449 to 1573534	100%	3,00E-40	100.00%	2550483 to 2550568	100%	7,00E-40	100.00%
assembly_3192681_3192403|ref_1506729_1507008|74|length:279	1506756 to 1507034	100%	6,00E-147	100.00%	1645913 to 1646191	100%	6,00E-145	99.64%
assembly_3256815_3256757|ref_727917_727975|88|length:58	727949 to 728006	100%	6,00E-25	100.00%	1153743 to 1153800	100%	1,00E-24	100.00%
assembly_3274701_3274944|ref_2313112_2313352|72|length:240	2313123 to 2313362	100%	2,00E-125	100.00%	1071980 to 1072219	100%	6,00E-125	100.00%
assembly_3274907_3275033|ref_1891982_1892108|77|length:126	1892009 to 1892134	100%	3,00E-62	100.00%	1424839 to 1424964	100%	3,00E-60	99.21%
assembly_3279160_3279828|ref_2833505_2834167|75|length:662	2833525 to 2834186	100%	0.0	100.00%	3767599 to 3768260	100%	0.0	99.70%
assembly_3279732_3279787|ref_1351634_1351689|91|length:55	1351659 to 1351713	100%	3,00E-23	100.00%	1805832 to 1805886	100%	6,00E-23	100.00%

Contig number	*Burkholderia mallei ATCC 23344* Position in the reference genome	Query Cover	E-value	Identity	*Burkholderia pseudomallei K96243* Position in the reference genome	Query Cover	E-value	Identity
assembly_3360360_3360260|ref_2289458_2289558|77|length:100	2289469 to 2289568	100%	6,00E-48	100.00%	1233823 to 1233922	100%	1,00E-47	100.00%

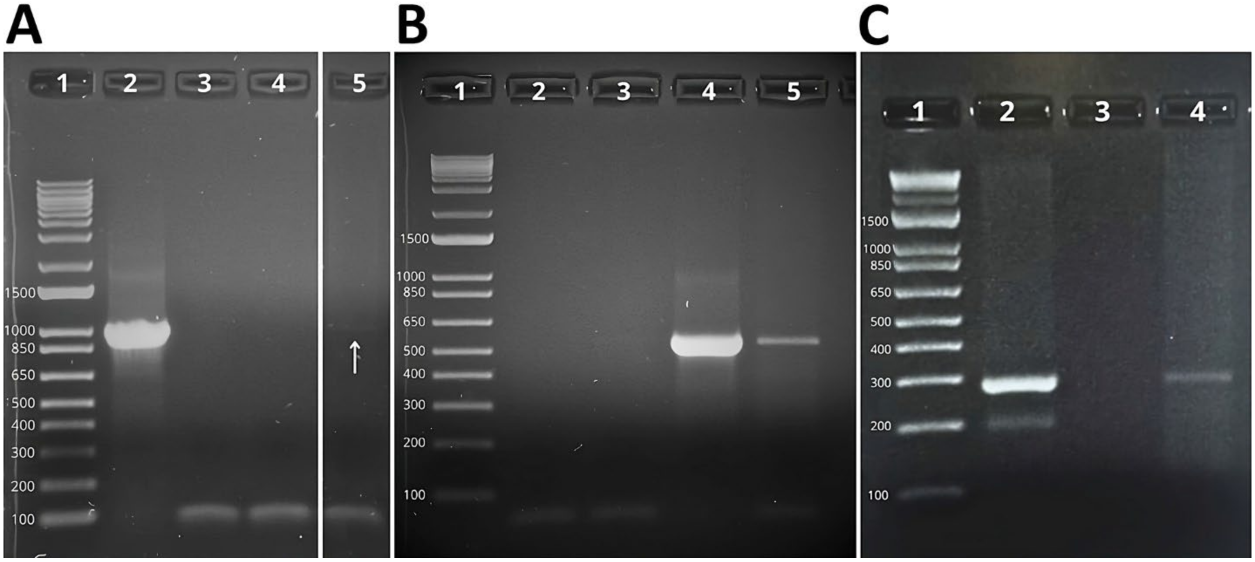

Appendix Figure 1. PCR results from DNA extracted directly from pleural drainage of a human patient in Brazil suspected of glanders caused by *Burkholderia mallei* - A) fliP-IS407A (a) (989 bp). Lane 1: 1 kb plus marker, Lane 2: Positive PCR control, Lane 3: Negative PCR control, Lane 4: Negative control for DNA extraction, Lane 5: DNA from pleural drainage. B) fliP-IS407A (b) (528 bp). Lane 1: 1 kb plus marker (Thermo Fisher, USA), Lane 2: Negative PCR control, Lane 3: Negative control for DNA extraction, Lane 4: Positive PCR control - DNA from *B. mallei* BAC 86/19, Lane 5: DNA from pleural drainage. C) MLVA marker (Bm17) (281 bp). Lane 1: 1 kb plus marker, Lane 2: Positive PCR control, Lane 3: Negative PCR control, Lane 4: DNA from pleural drainage.

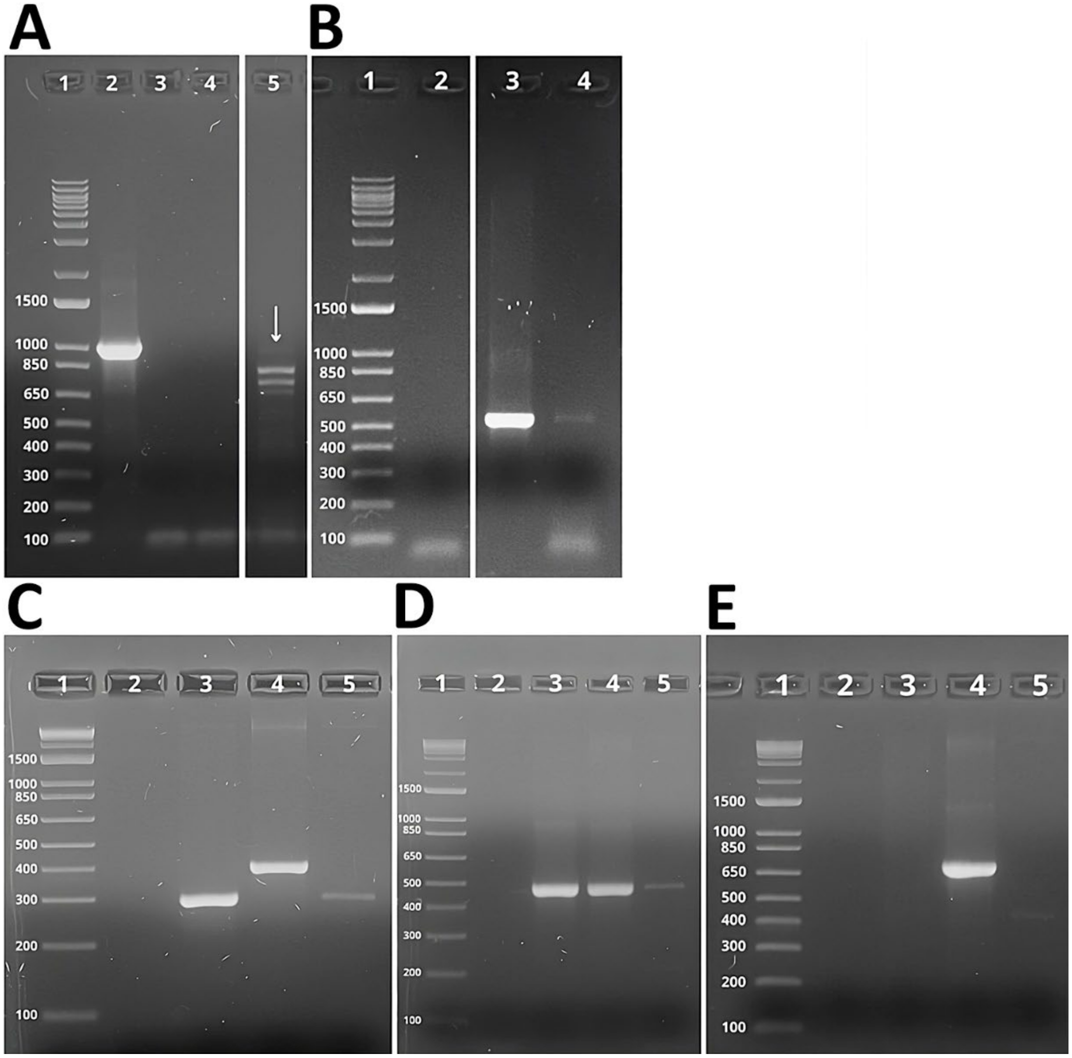

Appendix Figure 2. PCR results from DNA extracted from bacterial colonies obtained from the microbiological culture of the pleural drainage of a human patient in Brazil suspected of glanders caused by *Burkholderia mallei* - A) fliP-IS407A (a) of *B. mallei* (989bp). Lane 1: 1 kb plus (Thermo Fisher, USA); Lane 2: Positive control - DNA from *B. mallei* BAC 86/19, Lane 3: Negative PCR control, Lane 4: Negative control for DNA extraction, Lane 5: DNA from bacterial colonies. B) fliP-IS407A (b) of *B. mallei* (528bp). Lane 1: 1 kb plus, Lane 2: Negative PCR control, Lane 3: Positive control - DNA from *B. mallei* BAC 86/19, Lane 4: DNA from bacterial colonies. C) MLVA marker (Bm17) of *B. mallei* (281bp) or *B. pseudomallei* (321bp). Lane 1: 1 kb plus, Lane 2: Negative PCR control, Lane 3: Positive control - DNA from *B. mallei* BAC 86/19, Lane 4: Positive control - DNA from *B. pseudomallei*. Lane 5: DNA from bacterial colonies. D) Burk475 (475bp) of *B. mallei/B. pseudomallei*. Lane1: 1 kb plus, Lane 2: Negative PCR control, Lane 3: Positive control - DNA from *B. mallei* BAC 86/19, Lane 4: Positive control - DNA from *B. pseudomallei,* Lane 5: DNA from bacterial colonies. E) orf11 of *B. pseudomallei* (706 bp). Lane1: 1 kb plus, Lane 2: Negative PCR control, Lane 3: DNA from *B. mallei* BAC 86/19, Lane 4: Positive control - DNA from *B. pseudomallei,* Lane 5: DNA from bacterial colonies.
